# Supplementary material for: Evolution of a multifunctional trait: shared effects of foraging ecology and thermoregulation on beak morphology, with consequences for song evolution
Source: Proc Biol Sci. 2019 Dec 18;286(1917):20192474. doi: 10.1098/rspb.2019.2474 (PMC6939928; doi:10.1098/rspb.2019.2474)
Supplement: Figures and Tables [file rspb20192474supp1.pdf]

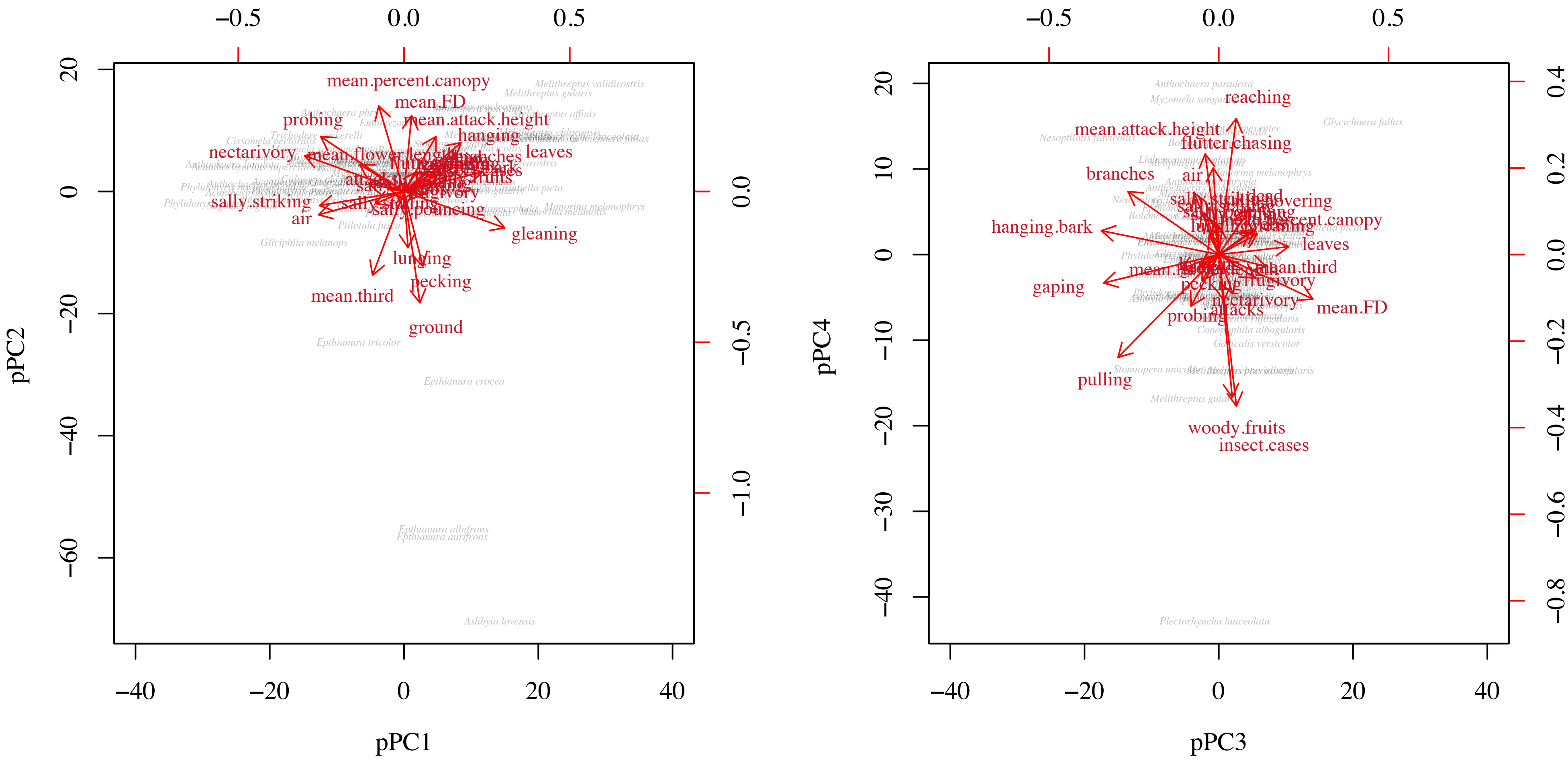

Figure S1: Biplots of phylogenetically-corrected Principal Component Analysis for observations of foraging behavior in honeyeater species. Species names are given at their PC value coordinates. PC loadings for each foraging variable are described as arrows.

PC3

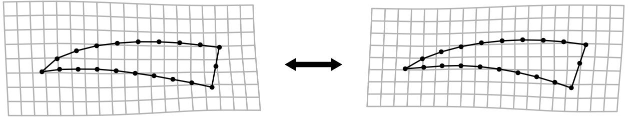

Figure S2: Warp grids illustrating the extremes of beak shape PC axis 3, which is described in the text as “tapering”.

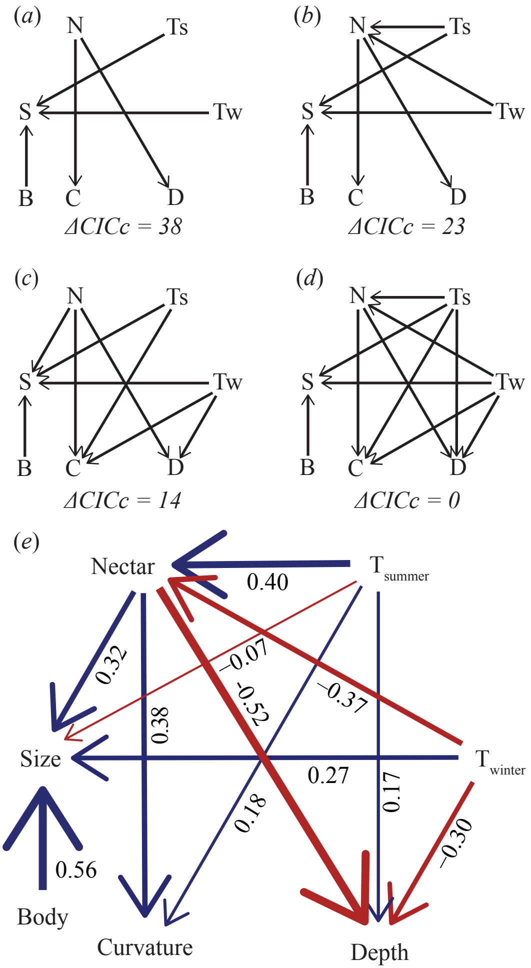

Figure S3: Phylogenetically-corrected path analyses were repeated using body size as a covariate. Format follows Figure 2. Model fit is assessed by the C-statistic information criterion (CICc) following [61]. The best fitting model is shown in (e), with red arrows indicating negative associations and blue arrows indicating positive ones; values shown below each arrow refer to correlation coefficients.

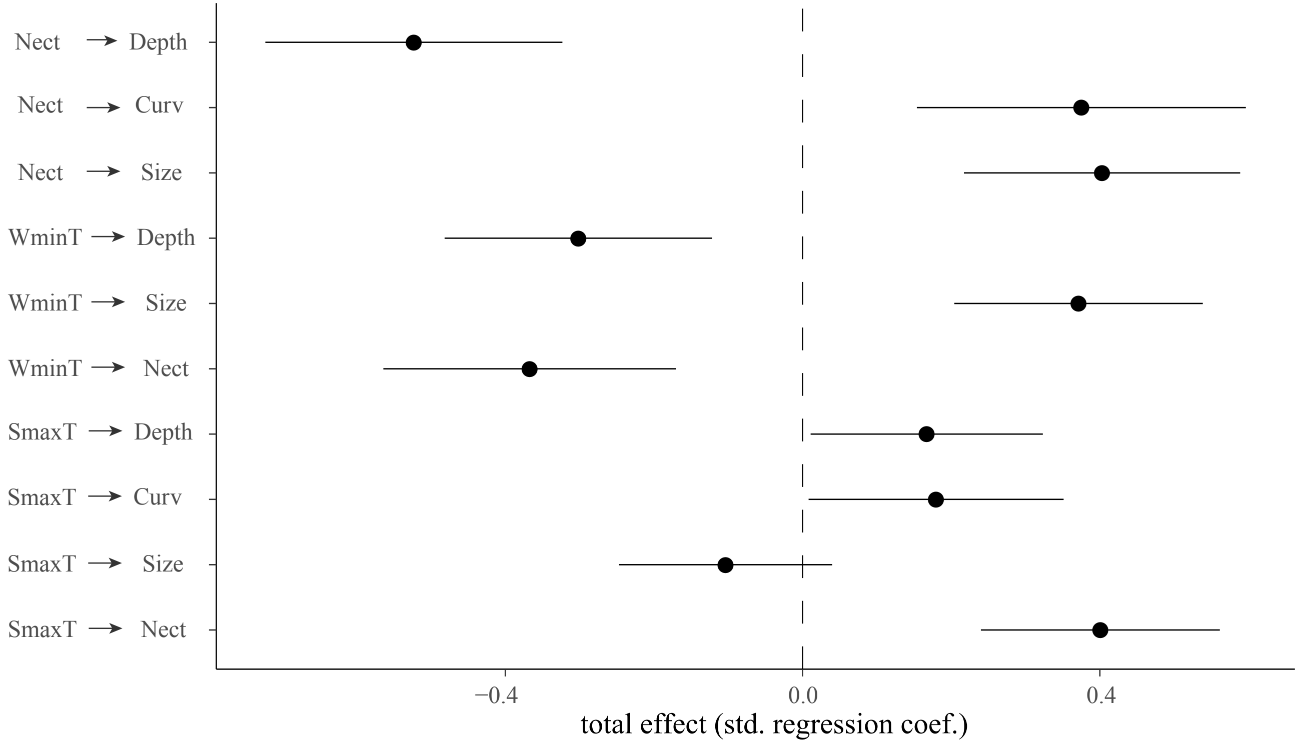

Figure S4: Effect size forest plot for total effect estimates from the phylogenetically-corrected path analysis described in Figure 2e. Error bars describe 95% confidence intervals around standardized regression coefficients. Abbreviations are as follows: Nect, Foraging pPC1; WminT, Winter Minimum Temperature; SmaxT, Summer Maximum Temperature; Depth, Beak Shape PC1; Curve, Beak Shape PC2; Size, Beak Centroid Size.

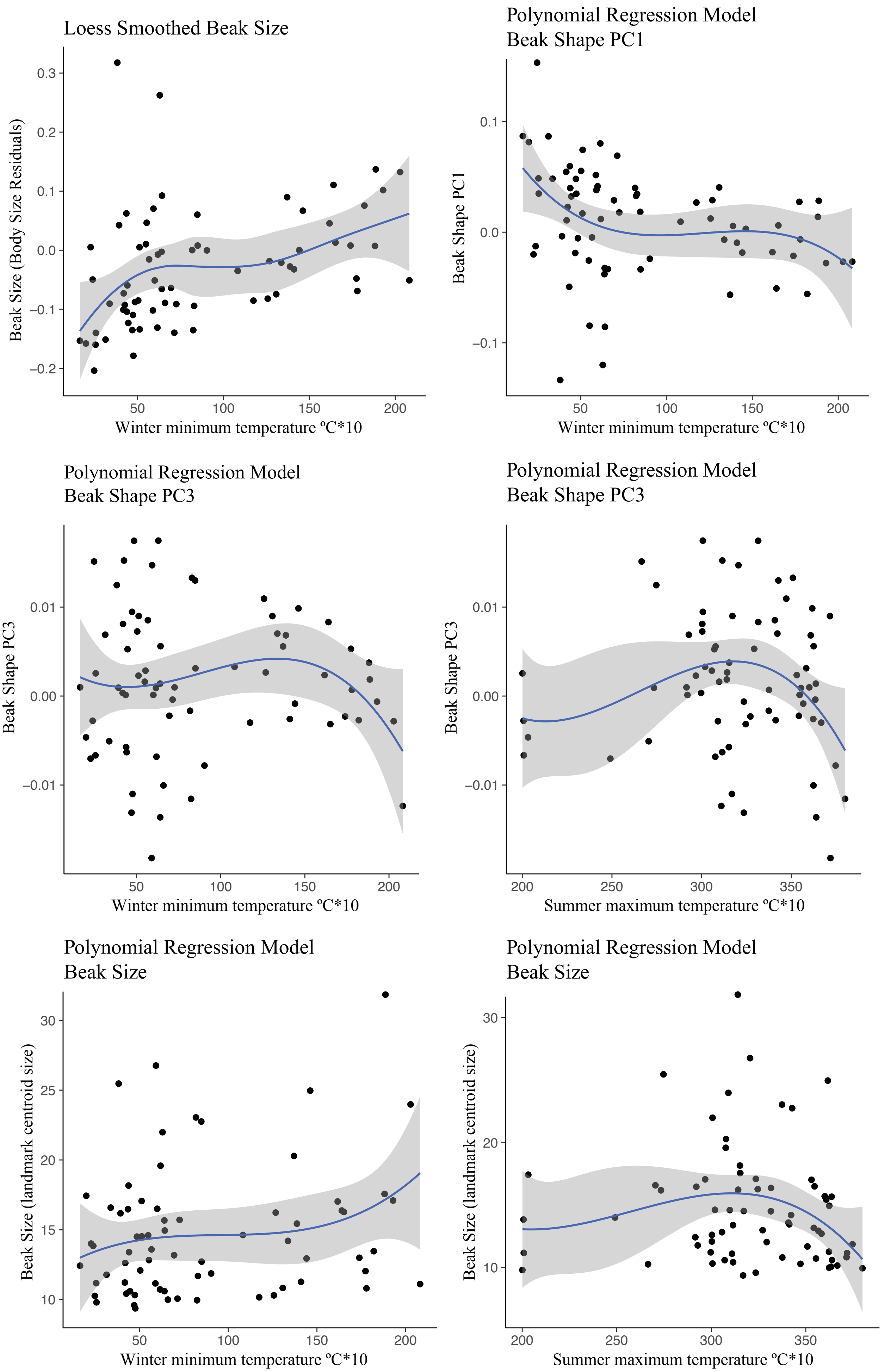

Figure S5: Polynomial regression models for the effect of winter and summer temperatures on beak shape and size. Model fitting was performed in multivariate, phylogenetically corrected framework, and relevant terms of best fitting models are shown here.

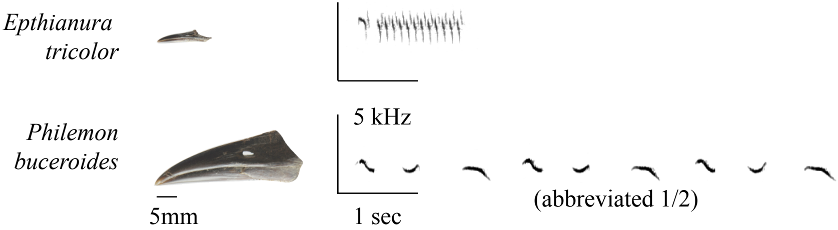

Figure S6: Comparison of extreme beak sizes and the songs those species perform. The Crimson Chat *Epthianura tricolor* is Australia's smallest honeyeater and performs a high trill, whereas the Helmeted Friarbird Philemon buceroides is Australia's largest honeyeater and performs a series of long whistles. Scale bars are shown for beak length (mm), time (seconds), and frequency (Hz).

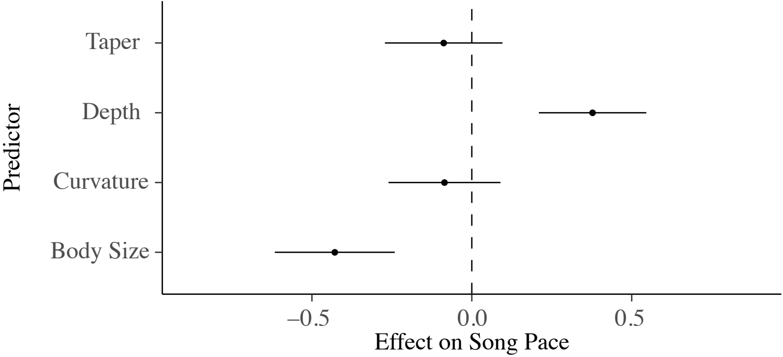

Figure S7: Forest plot for PGLS analysis results describing the effect of beak size and shape on song pace. Since beak size and body size are tightly correlated, we avoided including both as covariates. Here we repeat the analysis described in Figure 4a, panel 1 using body size rather than beak size.

Table S1. PCA loadings of ecological data describing dietary preferences and foraging behavior of honeyeater species, based on observations by ET Miller and SK Wagner (Miller et al. 2017). For each PC axis, descriptions are given for the three factors with the greatest absolute PC loadings. The first four PC axes together account for a cumulative 47% of the total variance.

PC1	Loading	PC2	Loading	PC3	Loading	PC4	Loading
Gleaning	0.86	Mean % Canopy	0.71	Pulling	-0.64	Reaching	0.61
Air	-0.73	Mean Third	-0.69	Gaping	-0.73	Woody Fruits	-0.64
Nectarivory	-0.87	Ground	-0.93	Hanging Bark	0.75	Insect Cases	-0.68

Table S2: Results of multivariate phylogenetic generalized least squares (PGLS) regressions conducted with each beak morphology response variable. Effect sizes and their standard errors are reported in terms of standardized beta regression coefficients.

Response Variable	Predictor Variable	Effect Size (std. B)	ES (std. err.)	t value	P value
**Beak PC1**	Foraging PC1 (17%)	0.485	0.101	4.785	0.000
(Depth)	Foraging PC2 (13%)	-0.046	0.121	-0.379	0.706
	Foraging PC3 (10%)	0.133	0.073	1.811	0.075
	Foraging PC4 (8%)	0.109	0.084	1.299	0.199
	Summer max T	0.169	0.092	1.836	0.071
	Winter min T	-0.308	0.092	-3.333	0.002
	Body Mass	0.110	0.097	1.140	0.259
**Beak PC2**	Foraging PC1	0.408	0.106	3.851	0.000
(Curvature)	Foraging PC2	-0.114	0.124	-0.919	0.362
	Foraging PC3	-0.122	0.089	-1.373	0.175
	Foraging PC4	-0.382	0.093	-4.116	0.000
	Summer max T	-0.264	0.113	-2.331	0.023
	Winter min T	-0.024	0.105	-0.231	0.818
	Body Mass	0.043	0.102	0.419	0.677
**Beak PC3**	Foraging PC1	0.148	0.124	1.196	0.236
(Tapering)	Foraging PC2	0.442	0.146	3.020	0.004
	Foraging PC3	0.238	0.099	2.401	0.019
	Foraging PC4	-0.206	0.106	-1.937	0.057
	Summer max T	0.059	0.127	0.464	0.644
	Winter min T	-0.265	0.120	-2.211	0.031
	Body Mass	-0.110	0.120	-0.921	0.361
**Beak Size**	Foraging PC1	-0.314	0.078	-4.038	0.000
	Foraging PC2	-0.033	0.095	-0.345	0.731
	Foraging PC3	-0.080	0.055	-1.453	0.151
	Foraging PC4	-0.045	0.064	-0.696	0.489
	Summer max T	-0.073	0.067	-1.089	0.280
	Winter min T	0.289	0.070	4.098	0.000
	Body Mass	0.567	0.075	7.572	0.000

Table S3: Results of AIC model fit comparisons among multivariate phylogenetic generalized least squares (PGLS) regressions conducted with polynomial temperature

Response Variable	Summer max T	Winter min T	delta AIC
**Beak PC1**	Linear	3rd Order	0.0
(Depth)	3rd Order	3rd Order	2.8
	Linear	2nd Order	4.1
	2nd Order	2nd Order	6.0
	Linear	Linear	9.1
	2nd Order	Linear	10.3
	3rd Order	Linear	12.1
**Beak PC2**	Linear	Linear	0.0
(Curvature)	Linear	2nd Order	1.2
	2nd Order	Linear	1.4
	Linear	3rd Order	2.7
	3rd Order	Linear	2.7
	2nd Order	2nd Order	4.3
	3rd Order	3rd Order	5.7
**Beak PC3**	3rd Order	3rd Order	0.0
(Tapering)	2nd Order	2nd Order	2.5
	2nd Order	Linear	3.8
	3rd Order	Linear	5.1
	Linear	2nd Order	5.8
	3rd Order	Linear	6.4
	Linear	Linear	6.4
**Beak Size**	3rd Order	3rd Order	0.0
	3rd Order	Linear	4.7
	2nd Order	2nd Order	6.5
	2nd Order	Linear	7.5
	Linear	3rd Order	9.9
	Linear	Linear	13.2
	Linear	2nd Order	14.8
